# Binding of aberrant glycoproteins recognizable by Helix pomatia agglutinin in adrenal cancers

**DOI:** 10.1002/bjs5.70

**Published:** 2018-04-27

**Authors:** R. Parameswaran, W. B. Tan, M. E. Nga, G. S. T. Soon, K. Y. Ngiam, S. A. Brooks, G. P. Sadler, R. Mihai

**Affiliations:** ^1^ Department of Endocrine Surgery National University Hospital Singapore; ^2^ Department of Pathology National University Hospital Singapore; ^3^ School of Biological and Medical Sciences, Oxford Brookes University Oxford UK; ^4^ Department of Endocrine Surgery Oxford University Hospitals NHS Foundation Trust Oxford UK

## Abstract

**Background:**

Aberrant glycosylation is a hallmark of cancer cells and plays an important role in oncogenesis and cancer progression including metastasis. This study aimed to assess alteration in cellular glycosylation, detected by lectin Helix pomatia agglutinin (HPA) binding, in adrenal cancers and to determine whether such altered glycosylation has prognostic significance.

**Methods:**

HPA binding lectin histochemistry was performed on archival paraffin wax‐embedded specimens of adrenocortical cancers excised from patients attending two tertiary referral centres. Benign tumours were used as controls. Demographic, histological and survival data were collected and compared between patients with HPA‐positive and HPA‐negative tumours.

**Results:**

Thirty‐two patients were treated for adrenal cancer between 2000 and 2016; their median age was 49 (range 23–79) years. Fifteen patients had functioning tumours (14 adrenal Cushing's tumours and 1 Conn's tumour). Mean(s.d.) tumour size was 127·71(49·70) mm. None of 10 control tumours expressed HPA‐binding glycoproteins. Invasion was associated with HPA‐binding glycoproteins (P = 0·018). Local recurrence or metastatic disease did not significantly differ between HPA‐positive and HPA‐negative adrenocortical cancers. Overall survival was significantly longer in patients with HPA‐negative tumours (median survival not reached versus 22 months in patients with HPA‐positive tumours; P = 0·002).

**Conclusion:**

Altered cellular glycosylation detected by lectin HPA is associated with poor survival in patients with adrenocortical cancer.

## Introduction

Adrenocortical cancers are rare endocrine tumours with an incidence of about 1–2 per million population. This disease appears to have a higher prevalence in women and is associated with extremely poor prognosis^1^, ^2^, ^3^, ^4^, ^5^. Factors that predict prognosis include stage of the disease and complete resection (R0) of the tumour^6^. Molecular markers of prognosis include Ki‐67 proliferation index above 10 per cent^7^, high steroidogenic factor 1 expression^8^, mutation of tumour suppressor gene *TP53*, and loss of retinoblastoma protein expression^9^.

Cell surface glycoproteins play an important role in cell recognition, signalling, proliferation and differentiation^10^. There are two main types of protein glycosylation: *N*‐linked (a *N*‐acetylglucosamine (GlcNAc) residue is added to the Asn residue within a consensus peptide sequence of Asn–X–Ser/Thr, where X can be any amino acid except proline) and *O*‐linked (a *N*‐acetylgalactosamine (GalNAc) residue is added to the hydroxyl group of Ser or Thr residue on the polypeptide) glycosylation.

Alteration in glycosylation is associated with invasive and metastatic potential in some cancer types^11^. Binding of the lectin *Helix pomatia* agglutinin (HPA) has been reported as a marker of altered *O*‐glycosylation in cancer. This has been shown in a wide range of human cancers including lung, breast, prostate, stomach, thyroid and colorectal cancer^12^, ^13^, ^14^, ^15^, ^16^, ^17^, ^18^. HPA recognizes a range of *O*‐linked glycans bearing the terminal monosaccharide GalNAc^19^, including blood group A substance, Forssman antigens^20^, Tn epitope^21^ and, to a lesser extent, terminal GlcNAc^22^. Studies on aberrant glycosylation in adrenocortical cancer have not been reported.

The aims of this study were to assess alteration in cellular glycosylation, detected by HPA binding in adrenal cancers, and to determine whether such altered glycosylation has prognostic significance.

## Methods

### Patient selection and follow‐up procedure

This was a retrospective study of patients who underwent surgery for adrenocortical cancer at two tertiary referral centres (Singapore and Oxford) between January 2000 and December 2016. Patients were eligible if archival paraffin wax‐embedded specimens were available and they had been followed up. Lectin histochemistry was performed to detect binding of HPA. All patients were followed until death or to the end of December 2016. Ethical approval was obtained from the institutional boards (IRB: 2014/00378; REC: 11/SC/0396).

### Preoperative investigation

All patients underwent two 24‐h urine biochemical evaluations to exclude the diagnosis of phaeochromocytoma by measuring 24‐h urinary metadrenalines, excessive secretion of steroids and their precursors, aldosterone and cortisol, to differentiate non‐functional from functional tumours. CT of the chest and abdomen was performed with contrast injection to assess characteristics of the tumour, lymph node involvement, adjacent organ invasion (of kidney, distal pancreas, spleen, liver or diaphragm), presence of intravascular thrombus in the inferior vena cava (IVC) or renal vein, and metastasis. If intravascular thrombus was suspected, further evaluation was performed with MRI. [^18^F]fluorodeoxyglucose PET was performed annually as part of follow‐up surveillance to detect metastasis or if patients had progression of symptoms. Both institutions followed similar protocols in terms of biochemical assessments and radiological investigations. In later years, however, PET was used more frequently for staging and assessment for metastasis.

### Demographic data

Demographic data, histological data, recurrence of disease, local invasion and mortality data were collected. Survival was estimated as overall survival (OS), defined as duration of survival from the date of surgery to the date of last follow‐up, and recurrence‐free survival (RFS), defined as the interval between surgery and diagnosis of recurrence for relapsed patients or from the date of surgery to date of last follow‐up for patients without recurrence.

### Lectin histochemistry

Lectin immunohistochemistry was performed on 5‐μm thick paraffin wax‐embedded clinical adrenal tumour samples using an avidin–biotin labelling technique validated by Brooks and Wilkinson^23^ to reveal binding of lectin HPA. For positive controls, sections of rat kidney, which shows strong and characteristic HPA labelling, were included in each labelling experiment. For negative controls, the lectin was omitted and the specificity of binding was confirmed by incubating the sections with HPA in the presence of 0·1‐mol/l GalNAc. Ten benign adrenal tumours were also assessed for controls.

Paraffin wax sections were dewaxed in xylene and rehydrated through a series of graded alcohols (100, 90 and 70 per cent (v/v)) to water. Endogenous peroxidases were then quenched by soaking the sections in 3 per cent (v/v) hydrogen peroxide in methanol for 20 min at room temperature before washing gently in running tap water for 5 min. The sections were then trypsinized in 0·1 per cent (w/v) trypsin (crude type II from porcine pancreas) and 0·1 per cent (w/v) calcium chloride in lectin buffer (0·6 per cent (w/v) Tris, 0·085 per cent (w/v) sodium chloride, 0·02 per cent (w/v) magnesium chloride, 0·01 per cent calcium chloride, pH 7·6) at 37^o^C for 20 min, in order to reveal lectin‐binding sites. The slides were washed in gently running tap water for 5 min after the trypsinization procedure. Biotinylated HPA was diluted in blocking buffer (5 per cent (w/v) bovine serum albumin in lectin buffer) at a concentration of 10 μg/ml. The slides were incubated with this solution for 1 h, washed in several changes of lectin buffer, and then incubated with 5‐μg/ml horseradish peroxidase conjugated avidin for 30 min. The sections were washed again in several changes of lectin buffer, and then lectin binding was revealed by incubation with hydrogen peroxidase/diaminobenzidine (DAB) (DAB Perioxidase Substrate Kit; Vector Laboratories, Burlingame, California, USA), prepared according to manufacturer's instructions, for 10 min. The sections were washed for 5 min under running tap water and then lightly counterstained in Mayer's haematoxylin. After blueing under running tap water, the sections were dehydrated through a series of graded alcohols (70 per cent (v/v), 90 per cent (v/v), 100 per cent), cleared in xylene, and mounted in DPX resinous mountant.

### Assessment of Helix pomatia agglutinin labelling

The guidelines described by Brooks and colleagues^24^ were used to classify the labelling of sections as positive or negative for HPA binding. Sections were scored as positive when 5 per cent or more of the cancer cells labelled positively for HPA binding and as negative when less than 5 per cent labelled positively. Assessments of HPA binding were performed by two observers who were blinded to the identity of the sample, and their results were compared. In cases of discordance, consensus was obtained after discussion. Interobserver variability was calculated by κ index using the formula: K = *P*o − *P*e/1 − *P*e, where *P*o is observed proportionate agreement and *P*e is overall random agreement probability.

### Statistical analysis

Statistical analyses were performed with SPSS® version 22.0 (IBM, Armonk, New York, USA). The association between positive HPA labelling and clinicopathological variables was analysed using the χ^2^ test and Wilcoxon signed rank test. Survival curves were determined by Kaplan–Meier analysis and compared with the log rank test. *P* < 0·050 was considered statistically significant.

## Results

### Patient and tumour characteristics

Forty‐seven patients were eligible for this study; 15 were excluded because they had no follow‐up (2) or tissue blocks were not available (13). Of the 32 included patients, 21 and 11 were operated on in Oxford and Singapore respectively; their demographic profile is shown in *Table* 1. Fifteen of the 32 tumours were functional and malignant (showed features of capsular and vascular invasion, extra‐adrenal extension, high mitotic figures, tumour necrosis and large size).

**Table 1:** Demographics of the study population

	No. of patients (*n* = 32)*
Age (years)†	49 (23–79)
Sex ratio (M : F)	15 : 17
Diagnosis	
Non‐functioning tumour	17
Cushing's tumour	14
Conn's tumour	1
Tumour size (mm)‡	127·71(49·70)
Tumour stage	
I	5
II	5
III	5
IV	17
Treatment	
Biopsy only	2
Adrenalectomy	13
Adrenalectomy + *en bloc* resection	17
Adjuvant treatment	
Mitotane only	22
Mitotane + other chemotherapy	4
Radiotherapy	2
Local invasion	
Yes	26
No	6
Metastasis	
Yes	14
No	18
Died	21

* Unless indicated otherwise, values are

† median (range) and

‡ mean(s.d.).

Treatment modalities for the patients were: surgery in 30 and adjuvant mitotane in 22. Four patients who received mitotane also underwent salvage chemotherapy with doxorubicin, cisplatin and doxorubicin. In patients who had surgery, 13 underwent adrenalectomy only and 17 had *en bloc* resection with nephrectomy (10), splenectomy (9), pancreatectomy (5) and IVC thrombectomy (4). Two patients did not undergo surgery and diagnosis was confirmed by CT‐guided biopsy. In 13 patients who underwent adrenalectomy only, malignancy was suspected in five cases but there were no features of local invasion on imaging warranting contiguous organ resection.

Based on the AJCC criteria, 17 patients had stage IV disease, and local invasion was observed in 26 patients. Fourteen of the 32 patients developed metastases, predominantly pulmonary. The next most common site of metastasis was the liver, followed by bone.

Median OS was 30 (range 1–168) months, RFS was 30 (6–156) months. The mortality rate for the cohort was 66 per cent (21 of 32) over the study period. Five of the 13 patients who had adrenalectomy alone developed local recurrence, and six of 17 had local recurrence following a radical *en bloc* resection.

### Helix pomatia agglutinin labelling of tissue samples

**Figure 1:**
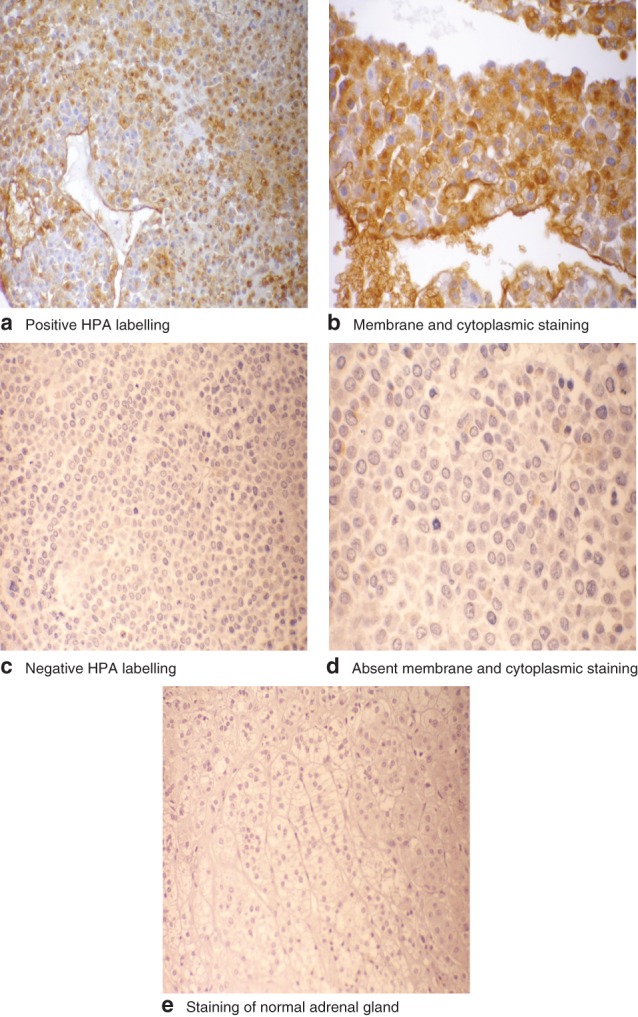
Results of Helix pomatia agglutinin (HPA) lectin immunohistochemistry in the tumours studied: **a** positive HPA labelling, as shown by intense deep brown staining, in adrenocortical cancer (ACC) (magnification ×10); **b** intense membrane and cytoplasmic staining of cancer cells (magnification ×20); **c** negative HPA binding in ACC with absence of any labelling (magnification ×10); **d** absent membrane and cytoplasmic staining of cancer cells (magnification ×20); **e** absent staining with HPA in a normal adrenal gland (magnification ×10)

Positive labelling for HPA binding was observed in 19 of the 32 patients. The difference in labelling between positive and negative tumours is shown in *Fig*. 1. Labelling was not observed in negative controls where HPA was omitted, or where HPA incubation took place in the presence of inhibiting GalNAc. The κ index between observers was 0·75, indicating satisfactory inter‐rater reliability for the immunohistochemical assessment of tumour samples.

### Correlation between clinicopathological variables

The correlation between the various clinicopathological variables and HPA labelling is shown in *Table* 2. No significant correlation was observed between HPA labelling and sex, histological diagnosis, tumour recurrence, stage of the disease, presence or absence of metastasis and adjuvant therapy. Of the 26 patients with features of local invasion, 18 had positive HPA labelling and eight negative HPA labelling (*P* = 0·018). Similarly, positive HPA labelling was seen in six of the ten patients with tumour recurrence (*P* = 0·961).

**Table 2:** Correlation between Helix pomatia agglutinin binding and clinicopathological variables

	Positive HPA binding (*n* = 19)	Negative HPA binding (*n* = 13)	*P* *
Sex ratio (M : F)	10 : 9	5 : 8	0·430
Tumour type			0·273
Cushing's tumour	10	4	
Conn's tumour	1	–	
Non‐functioning tumour	8	9	
Type of surgery			0·524
Biopsy only	1	1	
Adrenalectomy	8	5	
*En bloc* resection	10	7	
Tumour stage			0·451
I	4	1	
II	2	3	
III	2	3	
IV	11	6	
Invasion			0·018
Yes	18	8	
No	1	5	
Recurrence			0·961
Yes	6	4	
No	13	9	
Metastasis			0·618
Yes	9	5	
No	10	8	
Mitotane therapy			0·467
Yes	14	8	
No	5	5	
Weiss score			0·945
≥ 3	9	6	
< 3	10	7	
Died			0·007
Yes	16	5	
No	3	8	

HPA, *Helix pomatia* agglutinin.

* Log rank test.

### Significance of positive Helix pomatia agglutinin labelling

In univariable analysis, sex, type of surgery, stage, recurrence, metastasis, Weiss score and adjuvant therapy did not influence survival. Local invasion (*P* = 0·018) and positive HPA labelling (*P* = 0·007) were significantly correlated with survival (*Table* 2).

**Figure 2:**
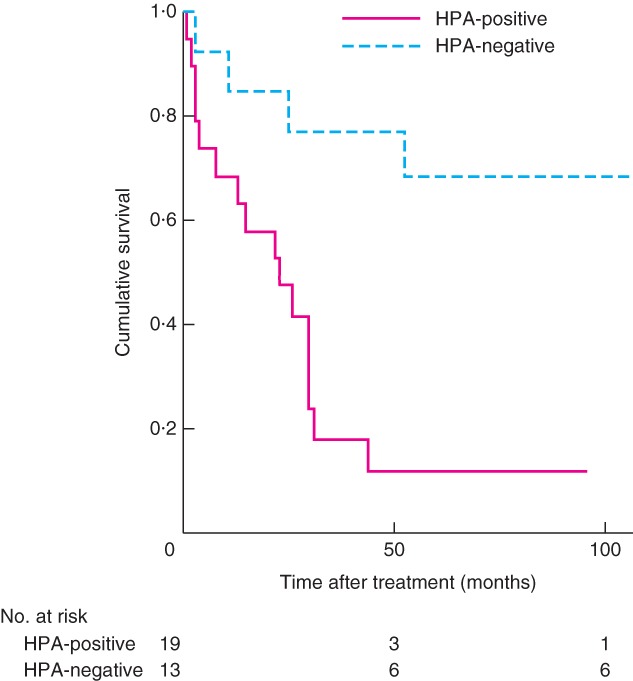
Kaplan–Meier survival curves for patients with positive and negative Helix pomatia agglutinin (HPA) immunolabelling. P = 0·002 (log rank test)

Differences in survival between patients with tumours that were positive or negative for HPA labelling are shown in *Fig*. 2. Patients with positive HPA labelling had a median survival of 22 (range 2–156) months; at the end of follow‐up, 16 of 19 had died. Of patients with negative HPA binding, all but three were alive at the end of the analysis period, with a mortality rate of 23 per cent (3 of 13); their survival was significantly better than that in patients with positive HPA labelling (*P* = 0·002).

## Discussion

This study has shown that aberrant glycosylation detectable by HPA lectin histochemistry is a predictor of poor survival in adrenal cancer. Glycosylation is the most common post‐translational modification of proteins and plays an important role in cell communication, interaction and adhesion^25^. Aberrant glycosylation is more likely to be seen in tumours with a more aggressive phenotype^26^.

In the present study, lectin cells showed significant cell membrane localization of HPA‐binding glycans where positive. The results were similar to those shown by Peiris and colleagues^18^ in metastatic colorectal cancer. The labelling pattern appeared to show an all‐or‐nothing effect: the cancers either labelled as clearly positive or completely negative. This is similar to the labelling pattern described by Brooks and Wilkinson in breast cancer^23^.

Adrenocortical carcinoma is an aggressive cancer with 5‐year survival rates of up to 60 per cent, with surgery the only effective curative treatment. Factors that affect survival include patient age, stage, completeness of resection, local invasion, functionality and metastasis^27^. In the present study there was no relationship between HPA lectin labelling and parameters including age, sex, histological type, surgery and chemotherapy. Positive HPA labelling was, however, associated with an increased risk of local invasion and significantly decreased median survival. Positive HPA labelling was not correlated with metastasis, unlike findings in studies of breast and colonic cancer^18^ ^28^. In studies that showed a correlation with metastatic state, the two main binding partners of HPA were identified as integrin α5 and α6, and annexin 2 and 4^18^ ^29^. These two binding partners were not evaluated in the present study. The exact binding partners in adrenal cancer are yet to be identified; characterization using affinity chromatography and mass spectrometry is required.

Markers such as Ki‐67 labelling index have been used to determine prognosis in adrenal cancer^7^ ^30^, ^31^ but continue to have significant variability in reporting amongst pathologists^32^. A recent publication^32^ on reporting variability described Ki‐67 as an unreliable marker. In the present cohort, any association between Ki‐67 and prognosis was not analysed as data pertaining to this variable were missing for several patients. HPA labelling was, in contrast, straightforward to interpret as positive or negative, making HPA lectin labelling a promising marker of prognosis in adrenal cancer.

Some studies^33^ ^34^ have shown that cortisol‐secreting adrenocortical cancers are associated with a poor prognosis. There was no difference in HPA binding or survival between functioning and non‐functioning adrenocortical cancers in the present cohort. It has been reported^35^ ^36^ that patients who received treatment with mitotane immediately after surgery had improved survival compared with those treated later after surgery, but another study^37^ did not show such a difference. The present study did not show a statistically significant difference in survival and HPA binding between patients who received mitotane and those who did not.

The limitations of this study include the small sample size with its inherent biases, the loss of 15 patients who could not be included in the study, and the retrospective design.

Positive HPA lectin labelling has the potential to be used as a marker of poor prognosis in patients with adrenocortical cancers, along with other currently used markers.
